# Boronic Acid-Based Approach for Separation and Immobilization of Glycoproteins and Its Application in Sensing

**DOI:** 10.3390/ijms141020890

**Published:** 2013-10-17

**Authors:** Xiaojin Wang, Ning Xia, Lin Liu

**Affiliations:** College of Chemistry and Chemical Engineering, Anyang Normal University, Anyang 455000, Henan, China; E-Mails: xjwanghxx@sohu.com (X.W.); liulin@aynu.edu.cn (L.L.)

**Keywords:** glycoproteins, boronic acid, separation, sensing

## Abstract

Glycoproteins influence a broad spectrum of biological processes including cell-cell interaction, host-pathogen interaction, or protection of proteins against proteolytic degradation. The analysis of their glyco-structures and concentration levels are increasingly important in diagnosis and proteomics. Boronic acids can covalently react with *cis*-diols in the oligosaccharide chains of glycoproteins to form five- or six-membered cyclic esters. Based on this interaction, boronic acid-based ligands and materials have attracted much attention in both chemistry and biology as the recognition motif for enrichment and chemo/biosensing of glycoproteins in recent years. In this work, we reviewed the progress in the separation, immobilization and detection of glycoproteins with boronic acid-functionalized materials and addressed its application in sensing.

## 1. Introduction

Glycoproteins play key roles in many biological processes, such as molecular recognition, inter- and intra-cell signaling, immune response, sperm-egg interaction, and regulation of development [1–3]. The occurrence of many diseases is accompanied by glycosylation of related proteins [4]. Thus, the analysis of their glyco-structures and concentration levels are increasingly important in diagnosis and proteomics [5,6]. However, the determination and characterization of glycoproteins are severely interfered with by other non-glycoproteins due to its inherent low abundance in complex biological samples. Therefore, efficient methods for isolation, enrichment and recognition of glycoproteins are indispensable. Glycoproteins can be commonly captured in a variety of ways, such as lectin affinity chromatography [7,8], solid-phase hydrazide capture [9,10], hydrophilic interaction chromatography (HILIC) [11,12] and so on. Among these methods, the lectin-based affinity method is one of the most popular techniques for the isolation and recognition of glycoproteins, but it only shows high selectivity towards *N*/*O*-linked glycoproteins. It is well known that boronic acids can covalently react with *cis*-diols to form five- or six-membered cyclic esters in an alkaline aqueous solution, while the cyclic esters dissociate when the medium is changed to acidic pH. This unique chemistry makes boronic acids attractive ligands for the many application of sensing, separation and self-assembly. In this work, we reviewed the progress in the separation, immobilization and detection of glycoproteins with boronic acid-functionalized materials and addressed its application in sensing.

## 2. Enrichment of Glycoproteins with Boronic Acid-Functionalized Materials

Currently, the methods applied to study glycopeptides or glycoproteins are more popularly based on mass spectrometry (MS) techniques [5,13–15]. However, because of their low abundances (2%–5%), MS responses of glycopeptides are severely suppressed by non-glycosylated peptides. Moreover, most glycosylation sites carry a multitude of glycans, giving rise to different glycoforms, reducing the relative amount of glycopeptides and making them difficult to detect. As a result, it is almost impossible to analyze substoichiometric glycopeptides without specific enrichment steps. The boronic acid-based method has gained increasing popularity because of its low bias, convenient enrichment, and avoidance of irreversible alterations of glycopeptides. To date, many ligands with boronic acid groups immobilized on monoliths, magnetic particles, mesoporous silica, polymer nanoparticles, gold nanoparticles and so on have been shown for the solid-phase extraction of glycopeptides. Current progress in the design and utilization of these materials for the separation of glycoproteins are addressed herein.

### 2.1. Boronic Acid-Functionalized Monoliths

Boronate affinity chromatography (BAC), based on the formation/dissociation of reversible covalent complexes between boronic acids and *cis*-diol in an alkaline/acidic aqueous solution, was developed for the trapping of glycosylated proteins [16–18]. Typically, polymer monolithic columns functionalized with boronic acid groups have been attractive in recent years because of their simplicity of preparation, versatile surface modification, higher permeability and good peak capacity [19]. Commonly, boronate-functionalized polymer monoliths can be synthesized by copolymerization and postpolymerization functionalization [20–22]. For example, Potter *et al.* was the first to report a boronate functionalized monolithic column synthesized by post modification on two base polymer monoliths [20]. Ren *et al.* prepared a boronate functionalized monolithic capillary column by *in situ* free radical polymerization of poly(4-vinylphenylboronic acid-co-ethylene glycol dimethacrylate, abbreviated as poly(VPBA-co-EDMA) [22]. These monolithic columns were evaluated with the separation of small *cis*-diol containing molecules. However, several issues may present serious obstacles for the wide application of these columns in the enrichment of glycoproteins. First, because reversed-phase interaction degrades specificity towards glycoproteins, organic solvents are required to be added to the mobile phase. However, for protein samples, the presence of organic solvents increases the risk of protein denaturation and precipitation. For this reason, Ren *et al.* synthesized a hydrophilic boronate functionalized polymeric monolithic column for the capture and separation of glycoproteins by the free radical polymerization through substituting the hydrophobic cross-linker EDMA with hydrophilic *N*, *N*-methylenebisacrylamide (MBAA) [23]. At the same time, Chen *et al.* reported the poly (3-acrylamidophenylboronic acid-co-ethylene dimethacrylate) (AAPBA-co-EDMA) monolith prepared in 530 mm capillaries by a one-step *in situ* polymerization procedure. The resulting boronate monolith was used as a sorbent for polymer monolith microextraction (PMME) and the extraction performance of this boronate monolith towards glycol-containing compounds (e.g., nucleosides, glycopeptides and glycoproteins) was examined [21]. Lately, Lin *et al.* reported the synthesis of hydrophobic poly (VPBA-co-EDMA) monoliths and addressed how it was applied to the selective capture of glycoproteins [24].

**Figure 1:**
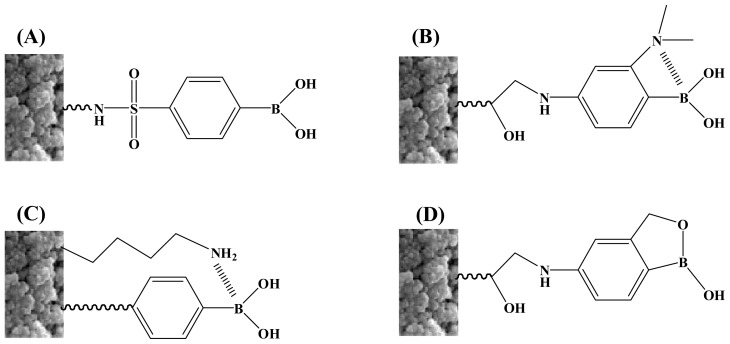
Boronic acid-based ligands on solid phase surface for diol binding at acidic pH.

Second, because the boronate ester is stable at alkaline pH, the chromatography in aqueous solution has to be performed in alkaline media. This may lead to the degradation of labile compounds. Thus, boronate functionalized monoliths that function at neutral pH were highly desirable for physiological samples. A conventional solution to this problem is to decrease the pKa value of the ligands by synthesizing novel boronic acids with exquisite structures through the introduction of an electron-withdrawing group, such as a sulfonyl or carbonyl group, into the phenyl ring (Figure 1A) [25–28] or the introduction of a neighboring amino group capable of boron–nitrogen (B–N) coordination into the ligand molecules (Wulff-type boronic acids) (Figure 1B) [29–31]. These two strategies can effectively improve the affinity around neutral pH; however, they share a common disadvantage that multi-step reaction routes must be employed to synthesize boronic acids that bring a reactive moiety for immobilization onto supporting materials. Recently, Ren *et al.* reported a new approach called ring-opening polymerization with synergistic co-monomers for the preparation of a boronate functionalized polymeric monolith that functions under neutral conditions (Figure 1C) [32]. This method obviates the synthesis and purification of a single functional monomer. However, the monolithic column failed to capture glycoproteins since they are associated with relatively low binding capacities. Further, Liu *et al.* prepared a novel functionalized material, called restricted access boronate affinity porous monolith for the capture of glycoproteins. This material showed high specificity for eight tested antibodies. But among seven non-antibody glycoproteins, lactoferrin, horseradish peroxidase (HRP), ribonuclease B (RNase B), α-acid glycoprotein (AGP), α-fetoprotein (AFP), erythropoietin (EPO), and ovalbumin (OVA), only AFP and OVA were retarded [33]. Benzoboroxoles are a unique class of boronic acids which have showed excellent water solubility and improved sugar binding capacity, even superior to Wulff-type boronic acid, in neutral water [34]. This class of boronic acids may provide an ideal solution to the obstacles mentioned above. Thereby, Li *et al.* synthesized a benzoboroxole functionalized hydrophilic polymeric macroporous monolithic column (Figure 1D) [35]. This boronate affinity monolithic column showed the best specificity and affinity towards cis-diol containing molecules among all boronate affinity monolithic columns, as well as high binding capacity under neutral or acidic conditions. These features overcome the disadvantages of the previous monolithic columns. However, this column only exhibited significant secondary separation capability towards nucleosides but not glycoproetins. So it is still necessary to explore new boronic acid-functionalized columns to resolve all these issues.

In addition, there are many other boronic acid-functionalized monolith materials for the selective separation of glycoproteins. For example, metal-organic gels (MOGs) have emerged as attractively alternative methods to form macroporous materials because the existence of macropores make monoliths tolerate a fast flow rate and easily achieve a high-throughput and high-efficiency separation of proteins. Yang *et al.* synthesized a macroporous boronate affinity monolithic column using a facile porogenic template of MOGs and applied it for the specific capture of glycoproteins [36]. In this work, the poly (3-acrylamidophenylboronic acid-co-ethylene dimethacrylate) [poly(AAPBA-co-EDMA)] monolithic column was synthesized by *in situ* free-radical polymerization in a stainless steel column, and a macroporous monolith was obtained after the systematic optimization of preparation conditions. The fabricated monolithic column was used to specifically capture glycoproteins, including horseradish peroxidase and transferrin. The column was also applied for a one-step purification of transferrin from a bovine serum sample, and the expected results were obtained. Recently, Lin *et al.* described a new approach for one-pot synthesis of organic-inorganic hybrid affinity monoliths by using the hydrolyzed tetramethyloxysilane (TMOS) and 3-methacryloxypropyltrimethoxysilane (g-MAPS) as co-precursors and 4-vinylphenylboronic acid (VPBA) as a functionalized organic monomer [37]. The resultant hybrid affinity monoliths exhibited specific recognition toward cis-diol containing biomolecules. Additionally, the practicability of the hybrid affinity monoliths was evaluated by using them as in-tube solid phase microextraction (in-tube SPME) for specific capture of glycoproteins from a real sample. Moreover, Lu *et al.* reported a pH manipulation strategy for fine-tuning the specificity of boronate affinity monoliths towards two sub-classes of glycoproteins, sialylated and nonsialylated glycoproteins [38]. When the binding pH is greater than the pKa of the boronic acid by one pH unit or more, the boronate affinity monolith preferentially binds to glycoproteins containing neutral sugars and excludes sialic acid containing glycoproteins due to electrostatic repulsion. When the binding pH is less than the pKa by one pH unit or more, the boronate affinity monolith binds to sialylated glycoproteins due to the exceptional binding affinity of the boronic acid towards sialic acid residues.

### 2.2. Boronic Acid-Functionalized Magnetic Particles

In recent years, the functionalization of various nanoparticles has shown great potential in immobilization, recognition, determination, separation, and enrichment of biomacromolecules. Magnetic particles, especially iron oxide nanoparticles, have attracted considerable interest due to their properties of good hydrophilicity, nontoxicity, biocompatibility and chemical stability. Surface modification of hybrid Fe_3_O_4_ nanocomposites with boronic acids has been used for glycoprotein separation because they provide a simple and fast procedure by using an external magnet. For example, in 2005 Lee *et al.* reported the capture and determination of glycoproteins using aminophenylboronic acid (APBA) modified magnetic beads [39]. The APBA layer was formed on the surface of carboxylic acid terminated magnetic beads by carbodiimide/*N*-hydroxysuccinimide-mediated amine coupling reaction. Glycoproteins, such as glycated hemoglobin (HbA_1c_), fibrinogen and RNase B, were separated and desalted using APBA magnetic beads with the help of an extra-applied magnetic field. The captured glycoproteins were then identified with matrix assisted laser desorption/ionizaton time of flight mass spectrometry (MALDI-TOF-MS) and confirmed again by peptide mass finger printing after digestion of proteins on magnetic beads by trypsin. At the same time, Sparbier *et al.* employed commercial concanavalin A- (ConA) and boronic acid-functionalized magnetic beads to isolate and enrich glycosylated peptides and proteins prior to MALDI-TOF-MS analysis [40], where carboxyl-functionalized magnetic particles were used to couple ConA and 3-aminophenylboronic acid onto the beads by using the carbodiimide method and polyglutaraldehyde method, respectively. The corresponding magnetic ConA beads and boronic acid beads showed specific binding toward the model proteins containing *N*-glycans of different oligosaccharide types and containing *cis*-diol groups, respectively. Lately, Zhou *et al.* reported the aminophenylboronic acid-functionalized magnetic nanoparticles for separation of glycopeptides and glycoproteins [41]. In this work, amine-magnetic nanoparticles were prepared by a one-pot solvothermal reaction and then modified with hexanedioyl chloride and 3-aminophenylboronic acid through a two-step amidation reaction. The specificity of the synthesized magnetic nanoparticles was evaluated by capturing of different model glycopeptides or glycoproteins (HRP and RNAse B) from mixtures containing non-glycomolecules (BSA and myoprotein).

The above boronic acid-modified magnetic particles can be applied to quickly separate and enrich glycopeptides or glycoproteins for proteomics. However, modification of boronic acid on magnetic particles requires a tedious multistep chemical reaction, which induces the decrease in the amount of boronic acid groups on magnetic particles and affects the enrichment efficiency for glycoproteins or glycopeptides. Gold nanoparticles (AuNPs) display easily tuned physical properties, including unique optical properties, robustness, and high surface areas. In the past few years, bi-functional Au-Fe_3_O_4_ nanoparticles, inheriting from the two components excellent surface chemistry, special optical properties, and superparamagnetic properties have been synthesized with various strategies [42–47]. Gold-coated magnetic nanoparticles, recognized as one of the major advances in nanobiotechnology, have been applied in the field of DNA hybridization assay and protein detection [48,49]. In view of the advantages of Au and magnetic particles, Qi *et al.* reported the synthesis of boronic acid-modified core-shell structure Fe_3_O_4_@C@Au magnetic microspheres and addressed its application in selective enrichment of glycopeptides and glycoproteins [50]. In this work, the Fe_3_O_4_@C magnetic microspheres were synthesized by two-step reactions including solvothermal and hydrothermal reactions. The Fe_3_O_4_@C@Au magnetic microspheres obtained by a self-assembly approach were modified with 4-mercaptophenylboronic acid (MBA) for capture of glycopeptides and glycoproteins.

As aforementioned, a basic operating pH is usually required for the capture of glycoproteins since boronic acids can form stable complexes with *cis*-diol compounds only in the dissociated form. This gives rise to the inconvenience of operation and the risk of degradation of labile molecules. The strategies relying on incorporating an electron-withdrawing group into the phenyl ring and incorporating an amine adjacent to the boronic acid to form an intramolecular boron–nitrogen coordination (Wulff-type) can effectively improve the affinity around neutral pH; however, these boronic acid compounds, if not commercially available, must be synthesized and purified through tedious procedures. For example, the synthesis of 4-(3-butenylsulfonyl)-phenylboronic acid and Wulff-type styrenic boronic acid need three and five steps, respectively. Recently, Liang *et al.* proposed a more general strategy, called self-assembled molecular team [51]. The central concept is that two molecules, a boronic acid and an amine, are assembled into a team with a configuration that the amine group is adjacent to the boron. As the amine coordinates with the boronic acid, the formed molecular team can function as a Wulff-type boronic acid, generating enhanced affinity at neutral pH.

In addition, Zhang *et al.* presented a novel and efficient method for the preparation of 3-aminophenylboronic acid-functionalized magnetite NPs via the click reaction between azide and alkyne groups [52]. The resulting click-Fe_3_O_4_@APBA NPs exhibited high adsorption capacity and excellent specificity towards glycoproteins. The selective isolation and enrichment of glycoproteins from egg white samples was successfully achieved. The other APBA-functionalized Fe_3_O_4_ NPs (nonclick-Fe_3_O_4_@APBA) were synthesized by conventional nucleophilic substitution reaction, for comparison of the adsorption of proteins on the two types of Fe_3_O_4_@APBA nanoparticles. Two glycoproteins, ovalbumin (OB) and transferring (Trf), and three non-glycoproteins, bovine serum albumin (BSA), lysozyme (Lyz) and horse heart cytochrome c (Cyt C), were chosen as model proteins. The as-synthesized click-Fe_3_O_4_@APBA NPs exhibited high adsorption capacity and excellent specificity toward glycoproteins and selective enrichment of target glycoproteins from real egg white samples as well. Lin *et al.* also prepared APBA-functionalized MNPs based on chemical co-precipitation and a multi-step covalent modification and evaluated its selectivity and binding capacity by testing glycoproteins (cellulose and ovalbumin) and non-glycoproteins (bovine hemoglobin, bovine serum albumin and lysozyme) as model samples [53].

### 2.3. Other Boronic Acid-Based Materials

**Figure 2:**
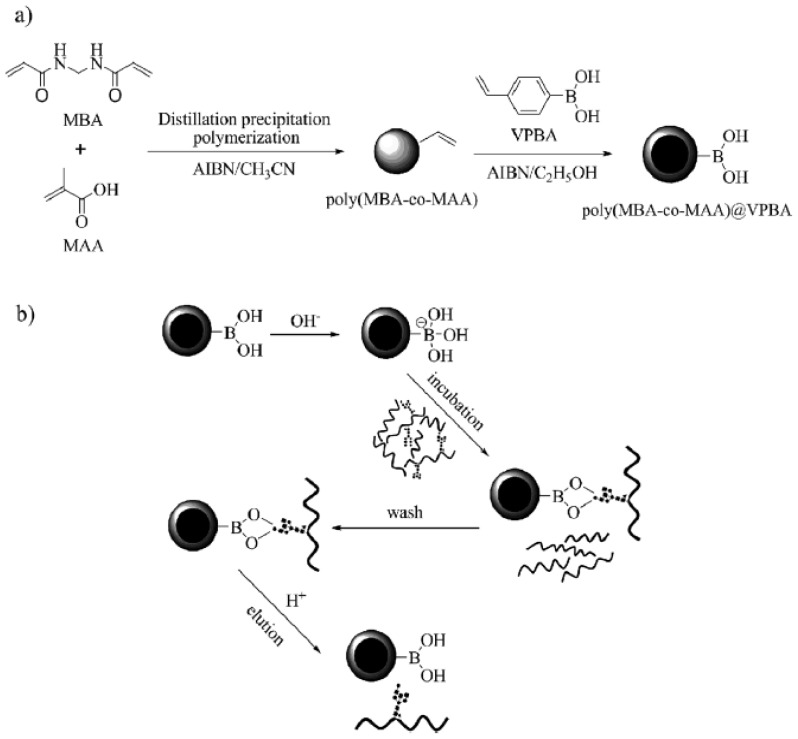
(**a**) Fabrication of poly(MBA-co-MAA)@VPBA nanoparticles and (**b**) application to the selective enrichment of glycopeptides. AIBN = 2,2-azobisisobutyronitrile, MAA = methacrylic acid, MBA = *N*,*N*-methyl-enebisacrylamide, VPBA = vinylphenylboronic acid. (From ref. [55], with permission. Copyright E 2012 John Wiley and Sons).

Polymer nanoparticles are presently attracting widespread interest in view of their merits of facile postmodification and good biocompatibility. Among various types of polymer nanoparticles, the core-shell morphology is one of the more advanced architectures. Recently, Shen *et al.* synthesized the core-shell boronic acid-functionalized nanoparticles SnO_2_@poly(2-hydroxyethyl methacrylate-*co*-styrene-*co*- 4-vinylphenylboronic acid (SnO_2_@Poly(HEMA-*co*-St-*co*-VPBA)) for selective enrichment of glycopeptides [54]. But in this work a multistep reaction is required for coating the VPBA monomer outside of SnO_2_. Furthermore, Qu *et al.* reported a facile and rapid method for the synthesis of core-shell boronic acid-functionalized polymer nanoparticles poly(*N*,*N*-methylenebisacrylamide-*co*-methacrylic acid)@-VPBA (denoted as poly(MBA-co-MAA)@VPBA)) for the enrichment of glycopeptides [55]. Such nanoparticles were composed of a hydrophilic polymer core and a boronic acid-functionalized shell designed for capturing glycopeptides (Figure 2). The hydrophilic core was formed with MBA and MAA by using distillation precipitation polymerization, and VPBA was introduced in the shell by free-radical polymerization rather than by employing a conventional solid-liquid heterogeneous grafting reaction. The results demonstrated that the synthetic poly(MBA-co-MAA)@VPBA nanoparticles might be a promising selective enrichment material for glycoproteome analysis.

The rational design of the surface chemistry of gold promotes specific interactions between the receptors and analytes. Mercapto terminated chain has been employed as the bridge that connects the base material and the functional groups through an Au-S interaction. For these reasons, gold-based plate techniques for fast enrichment of glycopeptides from peptide mixtures were also developed. For example, Tang *et al.* reported a MALDI target plate covered with an array of sintered AuNPs spots [56]. In this work, 4-mercaptophenylboronic acid was immobilized on the surface of AuNPs for the enrichment and assay of glycoproteins. The spots on the plate are used as a solid phase extractor to enrich target molecules and as a stable support of the MALDI matrix for the ionization of the retained molecules. The captured glycoproteins are further analyzed by MS. Because Au spots have large area ratio and Au can absorb UV laser to help desorption and ionization of trapped molecules, this *in situ* enrichment approach is effective and sensitive for analysis of glycopeptides from peptides mixtures. At the same time, Xu *et al.* also developed the boronic acid-functionalized Au-coated Si wafer (SiAuB) as MALDI target to enrich glycopeptides [57]. Recently, Zeng *et al.* reported a novel method for on-plate selective enrichment and purification of glycoproteins/glycopeptides [58]. This technique consists of a hydrophobic outer layer (F-SAM) and an internal boronic acid-modified gold microspot (900 mm). The outer hydrophobic layer can prevent the sample solution from diffusion and focus the sample in a small area, resulting in the enhancement in the detection sensitivity. The glycoproteins/glycopeptides are selectively captured through boronic acid covalently binding in the inner layer and the non-glycosylated proteins/peptides or high concentration salts can be removed by rinsing with 50 mM ammonium bicarbonate (NH_4_HCO_3_). This technique will be valuable for high-throughput analysis of low abundance glycoproteins/glycopeptides in complicated biological samples.

Because of the high surface area and large accessible porosity of mesoporous silica, boronic acid-functionalized mesoporous silica (FDU-12-GA) was synthesized by Xu *et al.* to enrich glycopeptides [59]. The synthesis of FDU-12-GA is quite simple under moderate temperature and neutral conditions and the large specific surface area greatly increases its binding rate, and the entire loading time needs only 15 min. In comparison to traditional direct analysis, this method enabled two orders of magnitude improvement in the detection limit of glycopeptides. With the plentiful diboronic acid function groups on the surface, no nonspecific binding is observed in the presence of non-glycosylated BSA protein.

## 3. Immobilization of Antibodies and Enzymes on Boronic Acid-Modified Substrates for Sensing

### 3.1. Immobilization of Antibodies for Sensing

**Figure 3:**
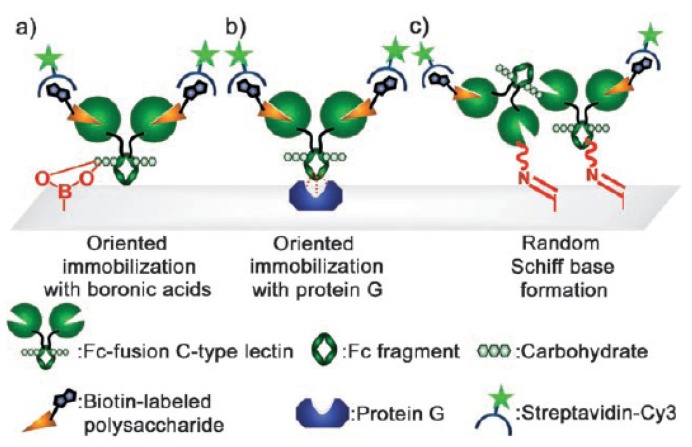
Illustration of the strategies used to generate microarrays of Fc-fused C-type lectin for detecting the biotin-labeled polysaccharide of G. lucidum: (**a**) Oriented covalent immobilization through boronate formation; (**b**) oriented noncovalent immobilization through protein G/Fc recognition; (**c**) random Schiff base formation. (For different Abs, the concentrations are different) (From ref. [60], with permission. Copyright E 2008 John Wiley and Sons).

A common approach towards developing immunoassays is to attach antibodies onto the surfaces of assay devices via a solid support. Traditionally, antibodies are modified at their lysine, arginine, aspartate, and glutamate residues by random amide bond conjugation or Schiff base formation, resulting in unsatisfactory activity in desired applications. Note that antibodies are glycoproteins belonging to the immunoglobulin superfamily, there are many reports on the immobilization of antibodies through the formation of boronate ester. Specifically, the carbohydrate moieties within the constant domain, fragment crystallizable (Fc), of the antibody can be covalently linked to a boronic acid-functionalized substrates. Lin and co-workers are the first to report the immobilization of antibodies on boronic acid-based surface [60]. In this conjugation approach, Fc-fused lectins were immobilized covalently on boronic acid-modified glass slides (Figure 3a). The binding activity of the immobilized lectins was compared to those of the products of noncovalent oriented immobilization by protein G (Figure 3b) and random covalent attachment by Schiff base formation (Figure 3c). The results indicated that the Fc-fused lectin microarray produced by boronate formation provides higher target sensitivity. To fabricate highly active immunoprobes for serum biomarker detection, they also reported the immobilization of antibodies on magnetic nanoparticles (MNPs) through the boronate ester formation and evaluated the performance by immunoaffinity extraction of multiple serum antigens [61]. Compared with the random immobilization of antibody on MNPs, the antibody self-oriented immunoprobe provides long-term stability (>2 months) and 5-fold extraction efficiency.

Lately, in Liu’s group, enzyme-conjugated carcinoembryonic and α-fetoprotein antibodies were immobilized onto 3-aminophenylboronic acid-modified SAMs gold electrodes for electrochemical sensing of carcinoembryonic and α-fetoprotein, respectively [62,63]. The attached enzyme-conjugated antibody on the electrode surface could catalyze the reduction of hydrogen peroxide in the presence of thionine. However, after the formation of the immunocomplex between antigen and the antibody, the access of the active center of HRP to thionine was partially inhibited, resulting in a decrease in the reduction current. With the similar way, they also developed an immunosensor for the detection of prostate specific antigen (PSA) [64]. Enzyme-conjugated prostate-specific antibody (HRP-anti-PSA) was immobilized onto the phenylboronic acid SAMs-covered gold electrode to catalyze the reduction of H_2_O_2_. After incubating an HRP-anti-PSA modified electrode in a PSA solution, immunocomplexes between HRP-conjugated anti-PSA and PSA formed on the electrode surface, which induced a decrease in the electrocatalytic response of H_2_O_2_ reduction. In 2010 Ho *et al.* compared the performances of two antibody immobilization methods on screen-printed graphite electrodes (SPGEs): the adsorption of monovalent half-antibody (monoAb) fragments of the anti-biotin Ab on AuNPs deposited SPGE through Au-S bond and the binding of the carbohydrate unit of the anti-biotin Ab with the 3-aminophenylboronic acid (APBA)-presenting SPGE surface [65]. The results demonstrated that the sensitivity of the Ab/APBA/SPGE biosensor was ca. 250 times higher than that of the monoAb/AuNPs/SPGE system. Recently, Moreno-Guzmán *et al.* reported the competitive electrochemical immunosensors for the detection of adrenocorticotropin (ACTH) and cortisol [66,67]. Through the interaction of boronic acids and glycosylation sites within antibodies, adrenocorticotropin (ACTH) and cortisol antibodies were immobilized onto aminophenylboronic acid-modified dual screen-printed carbon electrodes for the capture of biotin-ACTH and cortisol-alkaline phosphatase-labeled cortisol, respectively. The electroanalytical response was generated by using alkaline phosphatase-labeled streptavidin or alkaline phosphatase-labeled cortisol and 1-naphtyl phosphate as the enzyme substrate. The competitive binding between the native antigens and the labeled antigens to the immobilized antibodies induced the decrease in the amount of alkaline phosphatase. The developed immunosensor was further applied to a human serum sample with good results.

### 3.2. Immobilization of Enzymes for Sensing

**Figure 4:**
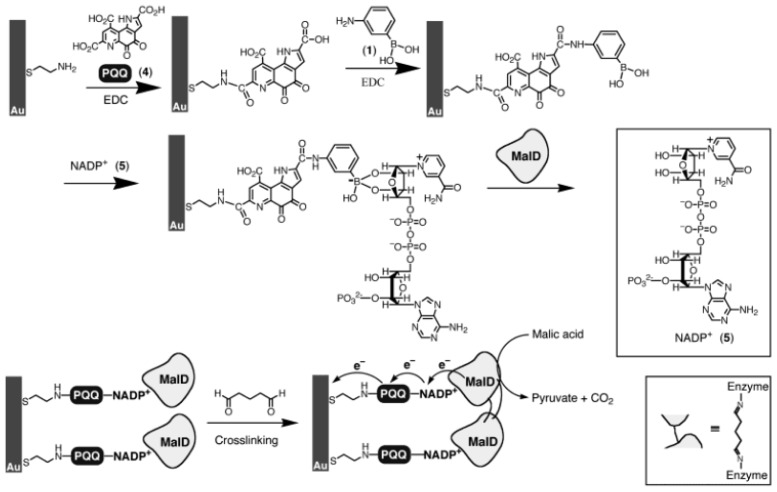
Assembly of a cross-linked-integrated malate dehydrogenase (MalD) electrode on a PQQ-phenylboronic acid-NADP+-functionalized surface. (From ref. [70], with permission. Copyright E 2002 American Chemical Society).

Enzyme immobilization is one of the key factors in constructing high-performance enzyme biosensors. Self-assembled monolayers (SAMs) are an inexpensive and versatile surface coating for molecular recognition for sensors and can reduce nonspecific adsorption of native proteins on metal substrate surface. In 2002 Abad *et al.* reported the covalent immobilization of glycoprotein horseradish peroxidase (HRP) through the direct binding of carbohydrate moieties to boronic acid groups on phenylboronates-oxyrane mixed alkanethiol SAMs [68]. The enzymatic electrodes were characterized by quartz crystal microbalance, atomic force microscopy, and electrochemical measurements. At the same time, Willner’s group introduced a method for the immobilization of flavoenzymes and NAD(P)+-dependent enzymes [69,70]. In their works, the FAD or NAD(P)^+^ cofactors were anchored onto the phenylboronic acid-modified gold electrodes for the capture of apoflavoenzymes (e.g., apo-glucose oxidase) or NAD(P)+-dependent enzymes (Figure 4), such as malate dehydrogenase (MalD) and lactate dehydrogenase (LDH). These results implied that boronic acid-diol interaction would be a highly effective binding for the immobilization of glycosylated enzymes. Recently, Ma *et al.* investigated the immobilization of glycosylated enzymes, such as glucose oxidase, horseradish peroxidase, dehydrogenase, on 4-aminomethylphenylboronic acid-modified glassy carbon electrode based on the interaction of boronic acid and carbohydrate moiety within the enzymes [71]. The adsorptions of three kinds of enzymes were investigated by cyclic voltammetry and electrochemical impedance spectroscopy. Besides, Liu *et al.* fabricated a phenylboronic acid self-assembled layer on glassy carbon electrodes for the recognition of glycoprotein peroxidase, where 3-aminophenylboronic acid is covalently bound to the electrochemical pretreated electrode surface with glutaraldehyde linkage [72]. The specific binding of glycoprotein peroxidase with the self-assembled layer was studied using horseradish peroxidase (HRP) as a model glycoprotein. Furthermore, they investigated the specific and non-specific binding of glycoproteins on the glassy carbon electrode covered with the thin film of poly(aniline boronic acid) [73]. Their results demonstrated that the specific interaction between boronic acid and glycosylation sites of the glycoproteins including HRP and glucose oxidase (GOx) is reversible since it can be released in acidic solutions and can also be split by sugars.

Nanomaterials have attracted more and more attention in the field of electrochemistry as electrode modified materials due to their high surface area, high adsorptivity and excellent catalytic capability. Electrodes modified with nanomaterials and boronic acids have therefore been developed for the immobilization of various enzymes. For example, Villalonga *et al.* reported the oriented immobilization of HRP for H_2_O_2_ detection on gold electrodes with an electropolymerized matrix of Au nanoparticles, followed by modification with 2-mercaptoethanesulfonic acid, 3-mercaptophenyl boronic acid and p-aminothiophenol [74]. Dong *et al.* reported a new method for the development of acetylcholinesterase biosensor based on specific binding between glycoprotein acetylcholinesterase and boronic acid-functionalized Fe@Au magnetic nanoparticles [75]. In this work, alginate-graphene composite-modified electrode was used for the attachment of boronic acid-functionalized Fe@Au magnetic nanoparticles through the interaction between the cis-diol of alginate and the boronic acid group on Fe@Au nanoparticles. Acetylcholinesterase was then immobilized via the bonding between the carbohydrate moieties within acetylcholinesterase and the boronic acid group on the nanoparticles. The immobilized enzyme retained relatively high bioactivity. The fabricated biosensor showed acceptable reproducibility, relatively good storage stability and high sensitivity and fast response to acetylthiocholine chloride.

**Figure 5:**
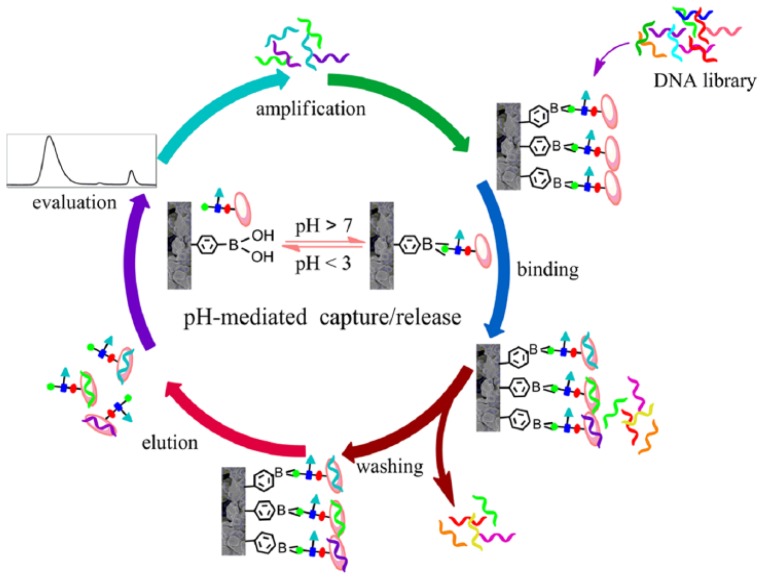
Schematic of boronate affinity monolithic capillary-based SELEX approach. (From ref. [76], with permission. Copyright E 2013 American Chemical Society).

Besides the above mentioned antibodies and glycated enzymes, other glycoproteins can also be immobilized onto boronic acid-modified surface for sensing. For example, Nie *et al.* reported a boronate affinity monolithic capillary-based systematic evolution of ligands by exponential enrichment (SELEX) approach for rapid selection of high-specificity glycoprotein-binding DNA aptamers [76]. In this work, the boronic acid moieties on the monolith were first saturated by the target glycoproteins by the boronate interaction (Figure 5). Then, a random ssDNA library was pumped into the monolithic capillary and incubated with the immobilized target for a certain period. Unbound ssDNA species were removed by rinsing the capillary with the DNA loading buffer. Bound species were eluted along with the target by an acetic elution solution and consecutively collected. After evaluation with capillary electrophoresis, desired fractions were combined and submitted to PCR amplification. The amplified species were sent for a new round of selection, and the process was repeated again and again until the binding affinity met the requirement or would not increase any more. The method endowed with rapid selection efficiency (only 6 rounds of selection or 2 days were needed) and high specificity toward the target glycoproteins overcomes the major drawbacks existed in conventional SELEX methods.

## 4. Detection of Glycoproteins by the Recognition of Boronic Acid-Functionalized Materials

The changes in the level of glycoproteins have been demonstrated to be associated with many diseases, such as inflammation and cancers [77,78]. Therefore, sensitive analysis of glycoproteins is keenly desirable for basic science advancement, clinical diagnostics and therapeutics. Based on the interaction between boronic acids and carbohydrate moieties within glycoproteins, various optical and electrochemical methods have been reported for the determination of glycoproteins. For example, De Guzman *et al.*, at the first time with surface plasmon resonance (SPR) spectroscopy, reported that glycoproteins (e.g., glycosylated avidin and RNAse B) can be captured by the carboxymethyl dextran substrate coupled with 4-[(2-aminoethyl)carbamoyl]phenylboronic acid (AECPBA) and readily released from the surface by borate buffer [79]. Several groups suggested that glycated hemoglobin (HbA_1c_), the most important index for a long-term average blood glucose level, can be immobilized on a boronic acid-covered electrode surface through the boronic acid-carbohydrate interaction [80–83]. It allowed HbA_1c_ to be detected indirectly by electrochemical techniques. Using spectral correlation spectroscopy and ellipsometry, Ivanov *et al.* reported that specific adsorption of mucin glycoprotein to the polymer brush containing phenylboronic acid caused an increase in the brush height by ca. 1.5 nm, which is indicative of the interaction of mucin and boronic acid [84]. Synthetic nanochannels have drawn enormous research attention because of their unique transport properties and potential applications in the field of biosensing, separation, and integration into nanofluidic devices [85]. Nguyen *et al.* and Sun *et al.* fabricated the 3-aminophenylboronic acid-modified biomimetic nanochannels for recognition of saccharide and glycoprotein ovalbumin [85,86]. Moreover, in mass spectrometric analysis, AuNPs are usually adopted in surface-assisted laser desorption/ionization mass spectrometry (SALDI-MS) or secondary ion mass spectrometry (SIMS) to obtain low LODs. Liu *et al.* reported a rapid and ultrasensitive strategy for glycoproteins detection with AuNPs as signal tags in laser desorption/ionization mass spectrometry (LDI-MS) analysis combined with boronic acid assisted isolation strategy [87]. Specifically, target glycoproteins were firstly isolated from sample solution with boronic acid-functionalized magnetic microparticles. Then, the 20-(11-Mercaptoundecanoyl)- 3,6,9,12,18-hexaoxaeicosanoicacid (MHA) modified AuNPs were covalently coupled to the glycoproteins through the carbodiimide-mediated amine coupling reaction. Finally, these AuNPs tagged glycoproteins were eluted from magnetic microparticles and applied to LDI-MS analysis and the mass signal of AuNPs was detected and recorded. However, all of these methods may not be regarded as specific for glycoproteins in blood because boronic acids can also bind to sugars and other glycated proteins [88–90]. Thus, the pretreatment of blood samples is required.

Sandwich-type immunoassay is a routinely used method for sensitive and selective detection of biomarker protein. In this format, a capture antibody against a specific target protein is first immobilized on a microtiter plate. After capture of protein from a sample solution, a labeled detector antibody is allowed to bind with the immobilized target. The protein concentration can then be determined by indirectly measuring the concentration of the probe attached to the detector antibody. Recently, our group developed a dual-amplified sandwich-type electrochemical biosensor for the detection of glycoproteins at low levels using 4-mercaptophenylboronic acid (MBA)-capped AuNPs (MBA-AuNPs) and dopamine (DA)-capped AuNPs (DA-AuNPs) [91]. The sandwich-type system was formed by specific capture of glycoproteins with receptor-modified electrode and recognition of glycoproteins through the formation of tight covalent bonds between the boronic acids of MBA-AuNPs and diols of glycoproteins. For signal read-out, electrochemically active DA-AuNPs were then anchored to the electrode surface via the interaction of boronic acids with catechol moieties on DA-AuNPs. This biosensor obviates the need of expensive and less stable antibody for the capture and recognition of glycoproteins and shows high sensitivity and selectivity.

**Figure 6:**
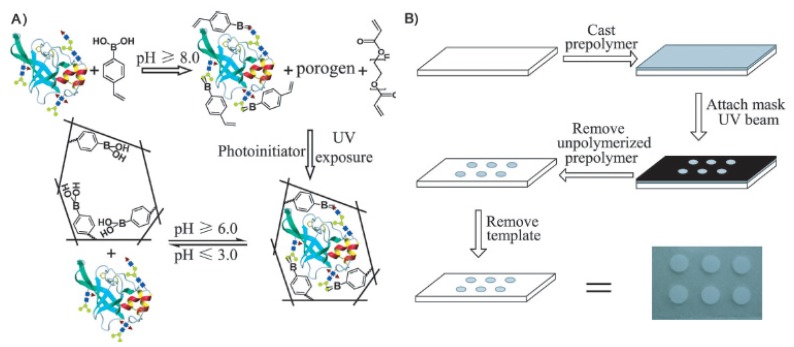
The principle (**A**) and procedure (**B**) of photolithographic boronate affinity molecular imprinting. (From ref. [93], with permission. Copyright E 2013 John Wiley and Sons).

Molecularly imprinted polymers (MIP), processed using the molecular imprinting technique which leaves cavities in polymer matrix with affinity to a chosen “template”, have found important applications, such as chemical separations, catalysis, or molecular sensors [92]. Recently, Li *et al.* developed a universal and facile approach for the imprinting and detection of glycoproteins using boronic acid-based UV-initiated polymerization and a photolithographic fabrication route (Figure 6) [93]. The generality of the approach was demonstrated with the successful imprinting of five distinct glycoproteins: HRP, RNase B, AFP, transferrin and anti-AFP monoclonal IgG. The use of a boronic acid as the functional monomer made the prepared MIP arrays feasible for the recognition of trace target glycoproteins in complicated real samples. Finally, the feasibility for real-world applications was demonstrated with an MIP array-based enzyme-linked immunosorbent assay (ELISA) of trace AFP in human serum. Moreover, Chen’s group prepared electrochemical-responsive Fe_3_O_4_@Au MIP nanofibers for the detection of glycoproteins [94]. The polymer nanofibers were first functionalized by introducing boronic acids and polymerizable double bonds with Fe_3_O_4_@Au NPs as the substrate. Then, glycoprotein HRP was directly covalently grafted on the surface of the functional NFs through the boronate interaction and polymerized via radical induced graft copolymerization.

**Figure 7:**
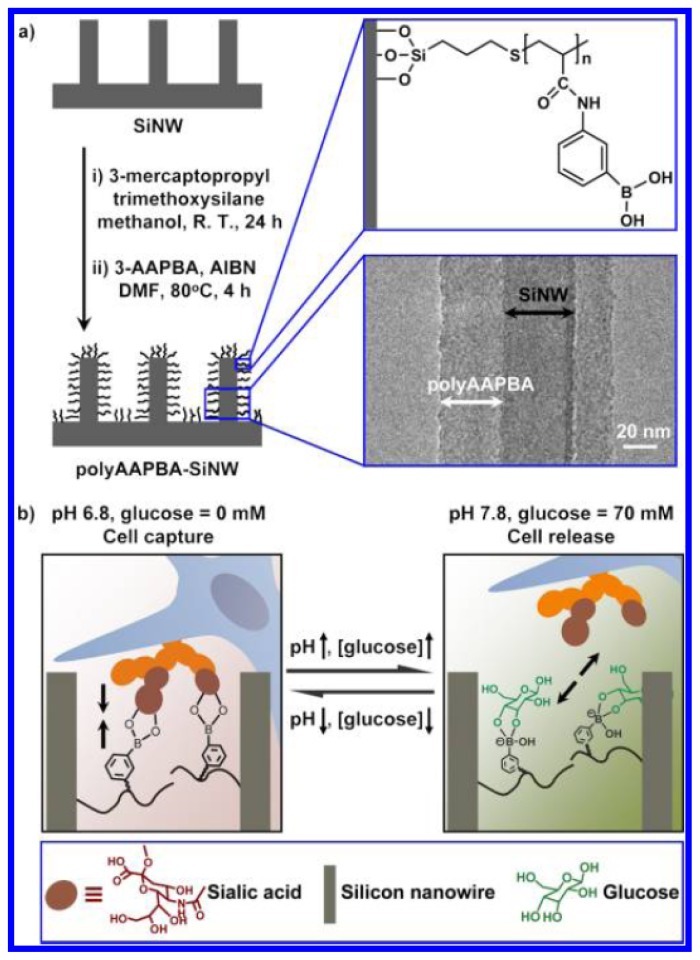
Schematic of the pH and glucose dual-responsive surface for cell capture and release. (**a**) Synthesis of poly(acrylamidophenylboronic acid) (polyAAPBA) brush on aligned silicon nanowire (SiNW) array. TEM image shows that the thickness of polyAAPBA is 30–40 nm (**bottom-right**); (**b**) Cell capture and release induced by pH and glucose. At pH 6.8 in the absence of glucose, the as-prepared surface captures targeted cells due to the specific binding between polyAAPBA brush on the surface and sialic acid existing in the membrane of the cells. By elevating the pH to 7.8 and increasing glucose concentration to 70 mM, competitive binding between tetrahedral anionic polyAAPBA and glucose leads to release of targeted cells. (From ref. [104], with permission. Copyright E 2013 American Chemical Society).

Besides the detection of glycoproetins, boronic acid-functionalized materials can also been employed for recognition and capture of cells by binding to glycoproteins in cell membranes. For example, sialic acids with a nine-carbon backbone are commonly found at the terminal position of the carbohydrate chains of glycoproteins and glycolipids in cell membrane. The unique distribution and ubiquitous existence of sialic acid on the cell membrane make it an important mediator in various biological and pathological processes and changes in sialic acid expression are closely associated with various disease states such as cancer, cardiovascular, and neurological diseases [95–100]. Increasing evidence has revealed that phenylboronic acid can form a stable complex with sialic acid in acidic medium below the pKa of PBA by the covalent binding between the diol function of overexpressed sialic acid and a phenylboronic acid [101]. Liu *et al.* report a new class of imaging probes based on semiconductor quantum dots with small molecular phenylboronic acid tags, which allow highly specific and efficient labeling of sialic acid on living cells [102]. Crich *et al.* demonstrated *in vivo* visualization of tumors by the highly selective dynamic covalent binding between overexpressed sialic acid and phenylboronic acid-targeting vector conjugated with an magnetic resonance imaging reporter [103]. Very recently, Liu *et al.* synthesized a pH and glucose dual-responsive surface that can efficiently and rapidly switch between capture and release of targeted cancer cells by grafting a poly(acrylamidophenylboronic acid) (polyAAPBA) brush from aligned silicon nanowire (SiNW) array (Figure 7) [104]. MCF-7 cells overexpressing sialic acid in the membrane was chosen as the model. At pH 6.8, polyAAPBA brush on the SiNW array can form specific binding with sialic acid expressed in the membrane of MCF-7 cell. With elevation of the pH to 7.8 and addition of glucose, a stable complex between tetrahedral anionic polyAAPBA and glucose replaces the polyAAPBA-sialic acid complex, inducing the release of MCF-7 cell. When decreasing pH to 6.8 without glucose, the tetrahedral anionic polyAAPBA is dehydroxylated and captures the MCF-7 cell again by specific binding with sialic acid. Such dual-responsive surfaces would find many biomedical and biological applications in cell-based diagnostics and *in vivo* drug delivery.

## 5. Conclusions

Boronic acid-based ligands and materials have displayed many applications in recognition and sensing of sugars. In recent years, the interaction of boronic acids and glycosylation sites within glycoproteins offers a unique selectivity profile, which allows for the efficient reduction of sample complexity. In conjunction with MS detection, boronic acid-modified materials can enhance the obtainable information both qualitatively and quantitatively by reducing matrix effects. The boronate interaction also allows for the straightforward and selective quantification of glycoproteins in complex matrices by optical or electrochemical detection. This review introduced different methods for separation, immobilization and detection of glycoproteins. We hope that readers will gain an appreciation of the wealth of potential for scientific discovery that remains in boronic-acid-mediated enrichment and sensing of glycoproteins.
